# Biotechnologically-Produced Myconoside and Calceolarioside E Induce Nrf2 Expression in Neutrophils

**DOI:** 10.3390/ijms22041759

**Published:** 2021-02-10

**Authors:** Kristiana M. Amirova, Petya A. Dimitrova, Andrey S. Marchev, Slaveya V. Krustanova, Svetlana D. Simova, Kalina I. Alipieva, Milen I. Georgiev

**Affiliations:** 1 Center of Plant Systems Biology and Biotechnology, 4000 Plovdiv, Bulgaria; amirova@cpsbb.eu (K.M.A.); andrey.marchev@yahoo.com (A.S.M.); 2 Laboratory of Metabolomics, Department of Biotechnology, The Stephan Angeloff Institute of Microbiology, Bulgarian Academy of Sciences, 139 Ruski Blvd, 4000 Plovdiv, Bulgaria; 3 Laboratory of Experimental Immunotherapy, Department of Immunology, The Stephan Angeloff Institute of Microbiology, Bulgarian Academy of Sciences, 26 Georgi Bonchev Str., 1113 Sofia, Bulgaria; petya_dimitrova@web.de; 4 Institute of Organic Chemistry with Center of Phytochemistry, Bulgarian Academy of Sciences, 9 Georgi Bonchev Str., 1113 Sofia, Bulgaria; Slaveya.Krustanova@orgchm.bas.bg (S.V.K.); Svetlana.Simova@orgchm.bas.bg (S.D.S.); Kalina.Alipieva@orgchm.bas.bg (K.I.A.)

**Keywords:** *Haberlea rhodopensis*, myconoside, calceolarioside E, phenylethanoids, Nrf2, NMR

## Abstract

The pathological manifestation of various diseases can be suppressed by the activation of nuclear factor erythroid 2 p45-related factor 2 (Nrf2), a transcriptional regulator of the cellular redox balance. *Haberlea rhodopensis* Friv. is a resurrection plant species endemic for Bulgaria, containing biologically active phenylethanoid glycosides that might possess antioxidant or redox activity. This study aimed to analyze the metabolic profile of in vitro cultured *H. rhodopensis* and to identify molecules that increase Nrf2 expression in bone marrow neutrophils. Fractions B, D, and E containing myconoside, or myconoside and calceolarioside E in ratios 1:0.6 and 0.25:1 were found to be the most active ones. Fraction B (200 µg/mL) improved neutrophil survival and strongly increased the Nrf2 intracellular level, while D and E, as well as, myconoside and calceolarioside E at the same ratios had a superior effect. Calceolarioside E (32 µg/mL) had stronger activity than myconoside, the effect of which was very similar to that of 2-cyano-3,12-dioxo-oleana-1,9(11)-dien-28-oic acid methyl ester (CDDO-Me), used as a positive control. These data indicate that both molecules, used alone or in combination have stimulatory activity on the endogenous Nrf2 level, indicating their therapeutic potential to regulate the cellular redox homeostasis oxidative stress-associated pathologies.

## 1. Introduction

*Haberlea rhodopensis* Friv. (family Gesneriaceae), commonly known as Rhodope silivriak or Orpheus’ flower, is a rare resurrection plant of the northern hemisphere spread all over Europe in the past and nowadays found only in the mountains of the Balkan Peninsula as a glacial relict from the Tertiary period. It is a native plant, endemic to Bulgaria and Greece. In Bulgaria, *H. rhodopensis* is distributed in Central and Southern Bulgaria, within Rhodope Mountain and some regions of Sredna Gora and Stara Planina Mountains. North-facing shady rocky slopes with high humidity at altitudes between 100 and 1700 m a.s.l. are the characteristic habitats of *H. rhodopensis* [1,2]. This plant species is included in the Red Data Book of Bulgaria in three categories, such as Rare species, Balkan endemic species, and Tertiary relicts, as well as, listed in the European Rare species category and in Appendix I of the Bern Convention [3]. A collection of *H. rhodopensis* is forbidden, as well as, its commercial exploitation [2]. Therefore, the development of *H. rhodopensis* plant in vitro systems could be a suitable alternative for its cultivation throughout the whole year in a controlled environment independently from climate changes, and for the biotechnological production of secondary metabolites, without further affecting plant natural habitats [4]. Indeed, the metabolite profiling, based on nuclear magnetic resonance (NMR) can be exploited as an effective approach to analyze *H. rhodopensis* extracts and to distinguish possible active molecules and standardized biomarkers in herbal preparations [5]. In our previous research, NMR-based metabolite profiling has been used to identify a specific group of molecules and compare their variations in different plant parts in *Sambucus ebulus* L. [6], *Rhodiola rosea* L. [7], and commercial preparations [8].

*Haberlea rhodopensis* is a poikilohydric, homoiochlorophyllous resurrection plant species. As a desiccation tolerant plant, it can survive long periods (up to 2 years) of extreme drought or desiccation (even less than 5% relative water content) and quickly restore its normal growth within hours of re-watering. During desiccation, it keeps approximately 80% of its chlorophyll contents. All these properties have turned *H. rhodopensis* into a model system to investigate its metabolic and photosynthetic processes, as well as, physiological stability when exposed to drought or freezing stress [1,9]. The main biologically active molecules identified in *H. rhodopensis* are the phenylethanoid glycosides myconoside and paucifloside, as well as, several flavone 8-*C*-glycosides, including hispidulin 8-*C*-*β*-galactopyranoside, hispidulin 8-*C*-(2”-*O*-syringoyl-*β*-glucopyranoside), hispidulin 8-*C*-(6-*O*-acetyl-*β*-glucopyranoside), and hispidulin 8-*C*-(6-*O*-acetyl-2”-*O*-syringoyl-*β*-glucopyranoside) [10,11]. Extracts or pure myconoside from the Orpheus’ flower have revealed a remarkable antioxidant and anti-aging activity [12], as well as, cytoprotective [13], radioprotective, anticlastogenic, and stimulatory effect on the regeneration of human fibroblasts in vitro [1,14]. Our previous expertise with phenylethanoid glucosides-rich extracts or pure molecules, such as verbascoside and forsythoside B, provided evidence on their anti-inflammatory activity on interferon gamma (IFN-γ) stimulated primary cultures of normal human keratinocytes [15]. In the last several years, two reports have shown that phenylpropanoids can act as activators of Nrf2 affecting Nrf2-ARE luciferase reports cell activity, Nrf2 protein level, and its downstream target genes expression [16,17].

Disrupted homeostasis, altered inflammatory response, apoptosis, proliferation, and metabolism can contribute to detrimental processes leading to pathological conditions [18]. Reactive oxygen species (ROS) are key elements of immune response but often have tissue deleterious effects when they are extensively generated and thus are found to be involved in the etiology of various diseases. The key regulator of ROS homeostasis is the nuclear factor erythroid 2 p45-related factor 2 (Nrf2) [19]. Structurally, Nrf2 possesses seven Neh domains and is bound to Kelch-like ECH-associated protein 1 (KEAP1) through its N-terminus Neh2 domain. At a normal cellular state, KEAP1 contributes to Nrf2 ubiquitination causing continuous Nrf2 proteosomal degradation [20,21]. Electrophiles and oxidants such as ROS can disrupt the KEAP1/Nrf2 interaction preventing Nrf2 ubiquitination and degradation. In turn, Nrf2 is directed to the nucleus and together with Maf proteins bind to the antioxidant responsible elements (ARE) regulating the transcription of various genes and contributing to improved redox balance, alleviated mitochondrial dysfunction, increased mitochondrial biogenesis, enhanced autophagy, and reduced inflammation. Therefore, the modulation of KEAP1/Nrf2 interactions by KEAP1 inhibitors or by direct Nrf2 activators can be used for therapy of ROS-related pathologies [22].

Herein, fractionation and isolation of myconoside and calceolarioside E from the crude methanolic extract of *H. rhodopensis* have been performed. The obtained fractions have been characterized and the pure molecules have been identified by NMR and further quantified by high performance liquid chromatography (HPLC). The biological effect on Nrf2 expression in primary bone marrow (BM) neutrophils of the fractions, containing different ratios of myconoside and calceolarioside E, as well as, single or combinatorial treatment of both molecules have been evaluated.

## 2. Results

### 2.1. Extraction and Isolation of Pure Compounds

**Figure 1:**
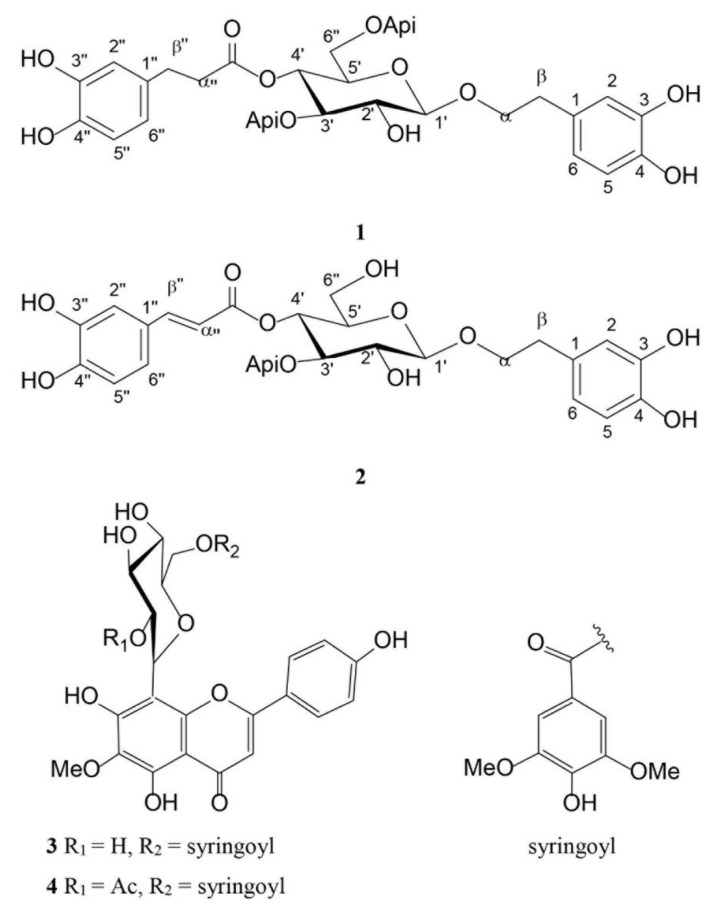
Structures of the compounds isolated from *Haberlea rhodopensis* aerial parts. (**1**) Myconoside; (**2**) calceolarioside E; (**3**) hispidulin 8-*C*-(2-*O*-syringoyl-*β*-glucopyranoside; (**4**) hispidulin fsali8-*C*-(6-*O*-acetyl-2-*O*-syringoyl-*β*-glucopyranoside).

*Haberlea rhodopensis* is known to contain the specific phenylethanoid glycosides myconoside and paucifloside, as well as, several flavone 8-*C*-glycosides [10,11]. During the development of the procedure for isolation of bioactive metabolites from *H. rhodopensis* aerial parts, we have obtained several fractions. Both, fraction B (1.86 g) and fraction C (0.39 g) contained predominantly the phenylethanoid glycoside myconoside along with a negligible quantity of unidentified compounds (**1**), while fraction D (0.026 g) and fraction E (0.134 g) were found to contain myconoside and the phenylpropanoid glycoside calceolarioside E (**2**) in ratios 1:0.6 and 0.25:1, respectively. Part of fraction E was used to isolate pure calceolarioside E (**2**). From fraction H (0.140 g), two flavone 8-*C*-glycosides were isolated, namely hispidulin 8-*C*-(2-*O*-syringoyl-*β*-glucopyranoside) (**3**) and hispidulin 8-*C*-(6-*O*-acetyl-2-*O*-syringoyl-*β*-glucopyranoside) (**4**), shown in Figure 1. The structures of the isolated compounds were determined by 1D and 2D NMR spectra.

### 2.2. Phytochemical Analysis

The phytochemical analyses of *H. rhodopensis* have been performed by NMR-based metabolite profiling and further by quantitative HPLC determination.

**Figure 2:**
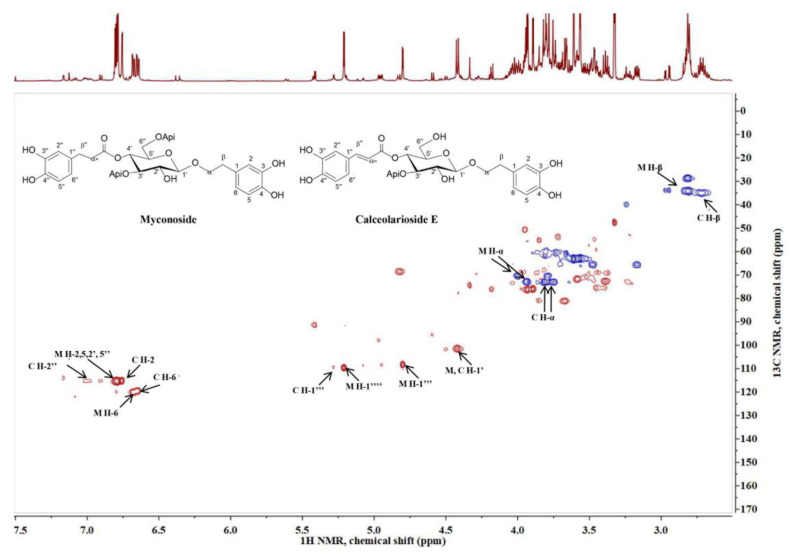
Heteronuclear single quantum coherence spectroscopy (HSQC) spectra of *Haberlea rhodopensis* extract and the characteristic signals of myconoside (M) and calceolarioside E (C).

Myconoside [*β*-(3,4-dihydroxyphenyl)-ethyl-3,6-di-*O*-*β*-D-apifuranosyl-4-*O*-α,*β*-dihydrocaffeoyl-*O*-*β*-D-glucopyranoside] consists of 3,4-dihydroxyphenyl moiety attached to the main sugar glucose, dihydrocaffeoyl structure linked to position C-4 of glucose and two *β*-apiosyl moieties linked to position C-3 and C-6 of glucose [23,24]. Calceolarioside E [1′-*O*-*β*-D-(3,4-dihydroxy-*β*-phenyl)-ethyl-4′-*O*-caffeoyl-*β*-D-apiosyl-(1′’’3′)-glucopyranoside] has a 3,4-dihydroxyphenylethyl structure also attached to the main sugar glucose, caffeoyl moiety linked to position C-4 of glucose, and one *β*-apiosyl structure linked to position C-3 of glucose [25]. The presence of myconoside and calceolarioside E in the extracts were confirmed first by their proton spectra. Additionally, the presence of both compounds was unambiguously confirmed through the proton-carbon single bond correlations observed in the heteronuclear single quantum coherence spectroscopy (HSQC) spectra (Figure 2), compared with authentic samples, and previously reported data [23,24,26,27].

The NMR signals corresponding to the structures of hispidulin 8-*C*-(2-*O*-syringoyl-*β*-glucopyranoside) and hispidulin 8-*C*-(6-*O*-acetyl-2-*O*-syringoyl-*β*-glucopyranoside) were in agreement with previously published data [10].

Furthermore, myconoside and calceolarioside E were quantified by HPLC in wild grown and biotechnologically cultivated *H. rhodopensis*. The purity of both molecules was estimated to be 94%. The content of myconoside and calceolarioside E in the wild grown plant were 6.51 ± 1.58 and 2.06 ± 0.34 mg/g dry weight (DW), while their amounts in the in vitro cultivated plant were 83.77 ± 5.09 and 62.22 ± 3.78 mg/g DW, respectively.

### 2.3. Studies for Biological Activity

**Figure 3:**
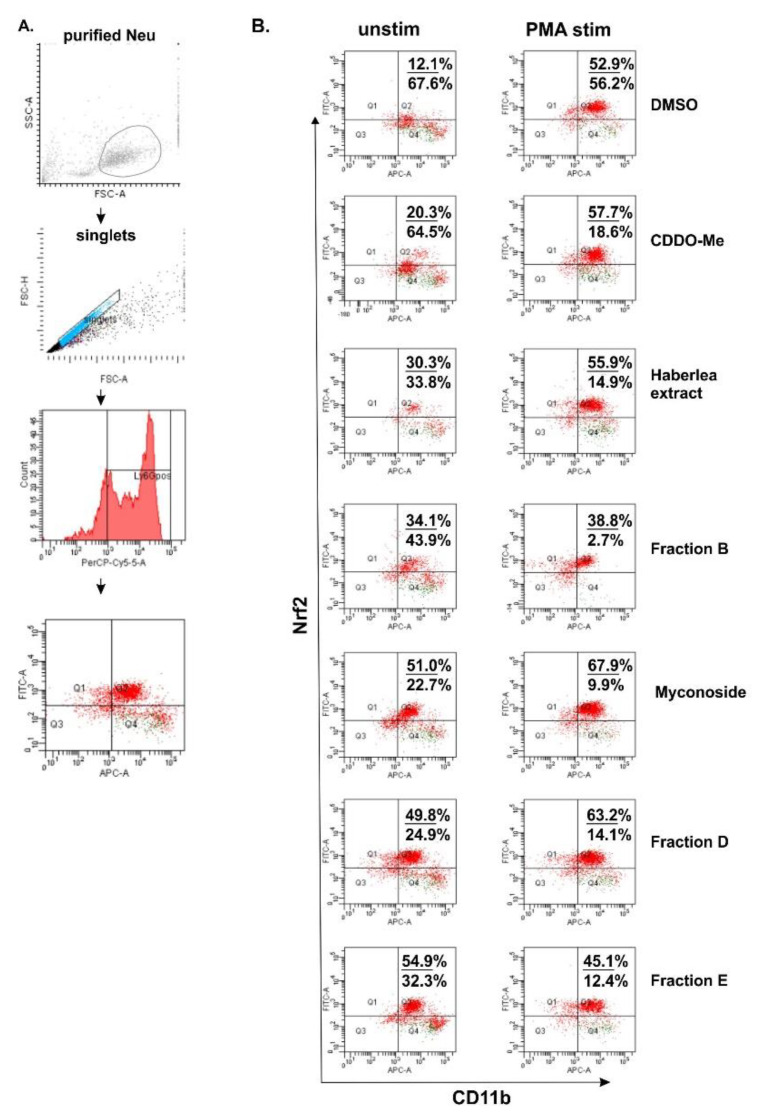
Flow cytometry analysis for the detection of endogenous nuclear factor erythroid 2 p45-related factor 2 (Nrf2) in bone marrow neutrophils. (**A**) Gating strategy involving the elimination of duplets. (**B**) Representative dot-plots showing the percentages of CD11b^+^ Nrf2^+^ neutrophils unstimulated or stimulated with phorbol 12-myristate 13-acetate (PMA) for 16 h and the analysis of collected 20,000 cells per sample. Dot-plot represents four quadrant images, Q1: CD11b^−^ and Nrf2^+^, Q2: CD11b^+^ and Nrf2^+^, Q3: CD11b^−^ and Nrf2^−^, and Q4: CD11b^+^ and Nrf2^−^. The numbers on the dot-plots indicate the frequencies of CD11b^+^ Nrf2^+^ cells/CD11b^+^ Nrf2^−^ cells.

Next, we studied the biological effect of *Haberlea* extract and fractions D and E on Nrf2 expression in BM neutrophils. According to the available databases, murine myeloid cells and mature neutrophils maintain a high overall steady-state activity of endogenous Nrf2 and strongly express Nrf2 [28]. In our study, we have isolated and stimulated BM neutrophils with phorbol 12-myristate 13-acetate (PMA) for 16 h in the presence of *H. rhodopensis* extract (200 µg/mL), fractions (200 µg/mL), myconoside (5 µM), or 2-Cyano-3,12-dioxo-oleana-1,9(11)-dien-28-oic acid methyl ester (CDDO-Me 5 µM), known as Nrf2 activator. In the utilized gating protocol, we have determined Ly6G^+^ neutrophils within the singlets gate (Figure 3A). Furthermore, we have analyzed the percentage of CD11b^+^ Nrf2^+^ positive cells within the gated Ly6G^+^ population (Figure 3A). We failed to detect CD11b negative cells and we noticed high and low CD11b expression on the neutrophils. In the control of dimethyl sulfoxide (DMSO)-treated unstimulated cells, we found the baseline Nrf2 level in 12.1% of CD11b^+^ neutrophils (Figure 3B). Regarding the treatment, the baseline intracellular Nrf2 expression in CD11b^+^ neutrophils increased upon the myconoside treatment. Indeed, this effect of myconoside on Ly6G^+^ CD11b^+^ Nrf2^+^ cells was stronger than that of the known Nrf2 activator CDDO-Me (Figure 3B). Notably, the extract, fraction B, D, and E, elevated the percentage of CD11b^+^ Nrf2^+^ neutrophils suggesting that they may contain compounds, inducers of Nrf2 expression. The main substance in fraction B was myconoside, while fractions D and E contain the phenylethanoid calceolarioside E at a specific proportion to myconoside. The results suggested a synergistic action of myconoside and calceolarioside E on the Nrf2 pathway and at the intracellular level defined as a 2.5-fold higher increase in Nrf2 expression in comparison to CDDO-Me (Figure 3B).

Neutrophil stimulation with PMA increased the percentage of CD11b^+^ Nrf2^+^ neutrophils in all the groups in comparison to the respective unstimulated groups. However, following the stimulation with PMA, myconoside and fraction D elevated endogenous Nrf2 to a greater extent than CDDO-Me (Figure 3B). Overall, the PMA stimulation of BM derived neutrophils can mask the compound action by triggering alternative pathways leading to a strong cytoplasmic Nrf2 expression [28,29].

**Figure 4:**
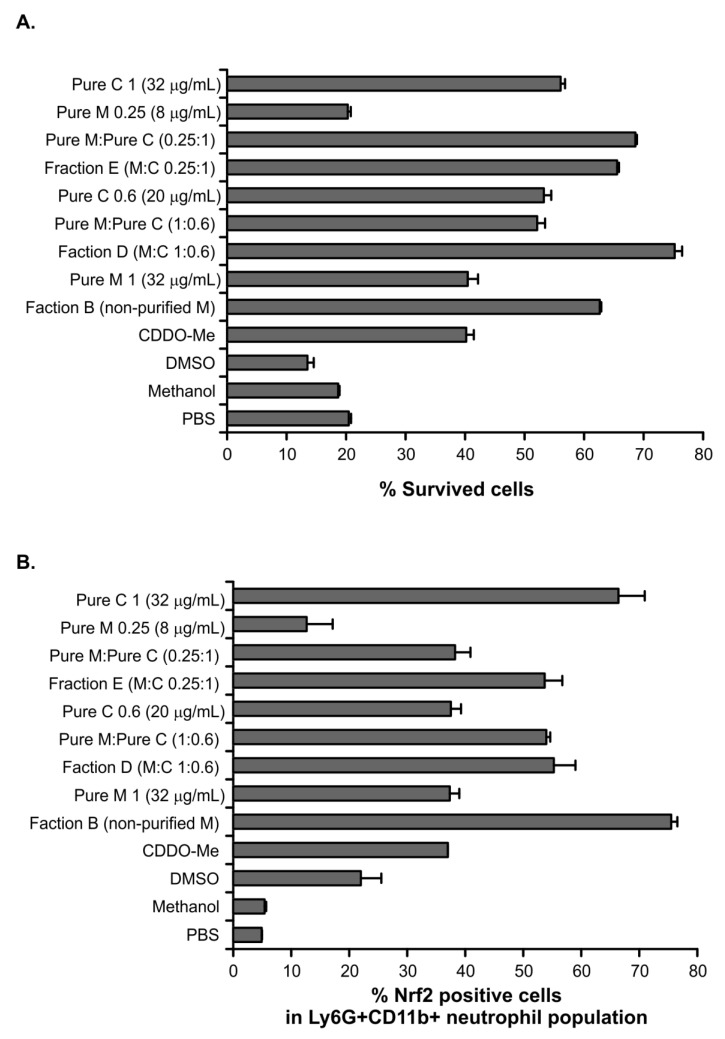
The effect of myconoside and calceolarioside E in the fractions on the percentage of viable (**A**,**B**) Nrf2^+^ cells in Ly6G^+^CD11b^+^ neutrophils. (**A**) The percentage of viable neutrophils was evaluated after 16 h of culturing in the presence of the fractions or pure compounds present alone or together at a particular proportion; myconoside (M) and calceolarioside E (C). The percentage of cell viability was calculated upon the Trypan blue exclusion of dead cells. (**B**) The percentage of Nrf2^+^ cells in mature neutrophils evaluated by flow cytometry. The data represent the mean ± standard deviation (SD) run in duplicates.

Herein, we observed that myconoside and fraction B both increased Nrf2 expression in BM neutrophils. Hence, fractions E and D showed a similar stimulatory effect on endogenous Nrf2 despite the fact that they contain different proportions of the main compounds myconoside and calceolarioside E. Therefore, we isolated and purified BM neutrophils and incubated them for 16 h with fractions B, D, E, and the pure compounds, both at concentrations corresponding to their proportion in the D and E fractions. The controls contained cells which are cultured with a phosphate buffered saline (PBS), DMSO (as myconoside is dissolved in DMSO), and methanol (as calceolarioside E is dissolved in methanol). We found that the cells incubated with fraction B (200 µg/mL) improved neutrophil survival and increased strongly the Nrf2 intracellular level (Figure 4). Pure myconoside at a concentration of 32 µg/mL, which was over six times lower than the content of myconoside in fraction B, failed to show such a strong effect on the percentage of viable or Nrf2^+^ neutrophils. Indeed, its action was similar to that of the known Nrf2 activator CDDO-Me (Figure 4). The concentration of myconoside was much higher than 32 µg/mL in fraction B (used at a concentration of 200 µg/mL in the bioactivity assays) indicating a dose-dependent increase in the percentage of Nrf2^+^ neutrophils by the compound (Figure 4B). Correspondingly, the low dose of myconoside 8 µg/mL induced Nrf2 expression in only 16% of neutrophils. Considering that the presence of myconoside in this fraction is 94%, the effect on the endogenous Nrf2 was not induced by the presence of other minor components in fraction B.

The analysis of fraction D showed that it contained the two main compounds myconoside and calceolarioside E in proportion 1:0.6. We have observed a better survival of neutrophils and increased percentages of Nrf2^+^ cells after 16 h of culture in the presence of fraction D in comparison to the known Nrf2 activator CDDO-Me. Indeed, pure compounds combined at the same proportion to that in fraction D affected the cell viability and endogenous Nrf2 level in a similar manner. Accordingly, the obtained data indicated that both myconoside and calceolarioside E have stimulatory action on Nrf2 intracellular expression (Figure 4B). Interestingly, the percentage of Nrf2^+^ cells after the treatment with fraction E, that contained calceolarioside E and myconoside at a ratio of 0.25:1, was similar to that of the fraction D-treated group (Figure 4B). Further, purified calceolarioside E (at a concentration of 32 µg/mL) was identified as a stronger inductor of endogenous Nrf2 in neutrophils than myconoside (at a concentration of 32 µg/mL).

Our data revealed that fractions D and E, which contained myconoside and calceolarioside E, as well as the pure compounds, itself, elevated the intracellular level of Nrf2. Biological consequences of high endogenous Nrf2 might be related to changes in ROS production, redox state, and/or to neutrophil recruitment from the BM and migration to the tissues. In two studies, the loss of Nrf2 was associated with an inability of neutrophils to migrate towards the wound or injury sites [28,29] and to mount an inflammatory response [30,31]. Considering these studies and our results, we suggest that the elevated Nrf2 expression in neutrophils by both compounds might be used for pharmacological modulation of several processes such as redox homeostasis, cell trafficking, and homing in pathological conditions.

## 3. Discussion

Myconoside is a phenylethanoid glycoside [10], distributed in several Gesneriaceae species, including *H. rhodopensis* [10,14,24] *Ramonda myconi* (L). Rchb., and *R. serbica* Panc. [24]. Calceolarioside E is a phenylpropanoid glycoside, isolated for the first time from *Lantana camara* L. leaves [32]. It is considered as a rare molecule, the presence of which is restricted to several plant families [24]. This compound has been identified in family *Episcieae* (*Episcia cupreata* (Hook.) Hemsl., *Nautilocalyx forgettii* (Spraque) Spraque, *N. lychee* (Hook. fil.) Sprague, *Alloplectus cristatus* (L.) Mart, *Columnea quercetin* Oerst, *Codonanthe carnosa* (Gardn.) Hanst, *Nematanthus wettsteinii* (Fritsch.) H. E. Moore [24] and family *Verbenaceae* (*Lippia alba* (Mill.) N. E. Brown [25], *L. radula* Sw. and *L. canescens* Kunth [33], *L. multiflora* Moldenke [34], *C. adscendens*, *C. foliosa* and *C. polifolia*) [35].

In the present study, the content of myconoside and calceolarioside E has been increased more than 12- and 30-folds, respectively in the in vitro cultivated plant compared to the wild grown *H. rhodopensis*. The ratio between myconoside and calceolarioside E was 1:0.3 in the wild grown *H. rhodopensis*, while in the in vitro cultivated plant this ratio changed to 1:0.7. The content of myconoside has been disclosed to greatly vary from 9 to 33% according to previous reports [11,14]. The content of calceolarioside E in *Lippia* spp., for example, has been estimated to be 0.08 mg/g DW [34]. Herein, we show that the in vitro cultured *H. rhodopensis* might be a valuable source of biologically active metabolites and that the expression of those metabolites was not compromised during the in vitro culturing. Hence, the production of the main compound myconoside can be further increased through genetic manipulation and/or upon changes in environmental factors (culture media composition, temperature, light, etc.) during the in vitro cultivation of *H. rhodopensis*.

Many plant-derived compounds can modulate the mechanisms of inflammation. To confirm that the pure compounds and *H. rhodopensis* fractions induce Nrf2 expression, we have utilized BM-derived neutrophils as an in vitro model system. Neutrophils are the first-responsive innate immune cells, since they are directly involved in injuries and inflammations [36] and therefore, are a potential target for the treatment of Nrf2-related therapeutic interventions. In the past decades, the most studied and promising plant-derived secondary metabolite with Nrf2-mediated anti-inflammatory activity was curcumin [37,38,39]. Other reports demonstrated that sulforaphane affected inflammation and neurodegenerative disease related to the Nrf2-KEAP1 axis. However, these compounds bound non-specifically and can embarrass the treatment effectiveness [40,41,42]. Therefore, the discovery of novel KEAP1 inhibitors or Nrf2 activators with improved selectivity is on demand. Within the present investigation, we have identified two natural substances that can modify the endogenous level of Nrf2, myconoside, and calceolarioside E. Their action on Nrf2 expression was observed after their application alone and when they were combined at particular ratios. Consequently, the selectivity of myconoside and calceolarioside E to bind directly to the Nrf2 molecule is worth further in-depth investigation. However, we provide clear evidence that the fractions and the purified compounds from in vitro cultured *H. rhodopensis* might be valuable Nrf2-modifying agents with a therapeutic potential.

Numerous studies revealed that myconoside is a potent antioxidative modulator [13]. Extracts from *H. rhodopensis* have an immunomodulatory potential through ROS reduction in radiation-exposed rabbits [43], and mimic the ROS-induced apoptotic effect in the non-malignant HEK293 cell line and prostatic cancer cell line PC3 [44].

Calceolariosides, such as calceolarioside B have promising anti-respiratory syncytial virus effects [45]. Calceolarioside attenuated doxorubicin-induced cardiotoxicity in H9c2 cells via the upregulation of antioxidant enzymes and suppression of apoptosis [46]. Those studies supported our interest towards investigating the biological activities of calceolariosides.

Here, we found that myconoside and calceolarioside E in ratio 1:0.6 can accelerate Nrf2 expression in neutrophils. As a master regulator of cellular responses, Nrf2 is modulating the levels of oxidative stress and influencing the course of diseases caused by oxidation. Additionally, the activation of Nrf2 can be essential for neutrophil recruitment and accumulation at the damaged sites as it has been demonstrated in skin during contact hypersensitivity [29].

Myconoside possess an additional *β*-apiosyl moiety at the C-6 position of the glucose ring compared to calceolarioside E. Apart from that, calceolarioside E at the C-4 position is linked to the dihydrocaffeoyl structure, while myconoside possess caffeoyl moiety at the same position. Our bioactivity data revealed that when used alone at the same concentration (32 μg/mL), myconoside and calceolarioside E induce different responses in neutrophils on both cell viability and Nrf2 expression. Accordingly, we could speculate that both the extra double bond in the calceolarioside E molecule and the additional apiosyl ring in myconoside are important for the induced biological effects. Moreover, when combined in the ratios identified in the isolated fractions D and E, the two phenylethanoids induce greater Nrf2 abundance in neutrophils in a synergistic manner. This further supports the notion that despite their structural similarity, myconoside and calceolarioside E induce Nrf2 activation to a varying degree and the underlying mechanisms are yet to be clarified. Similarly, in our previous report, we provide evidence that regardless of their close structural similarity, the two phenylethanoids verbascoside and isoverbascoside modulate inflammation through different pathways and to a different degree [47]. Several reports also demonstrated that phenylethanoid glycosides, such as salidroside, verbascoside, isoverbascoside, and echinacoside activate Nrf2 expression at concentrations between 0.1–10 µg/mL and hence, affect H_2_O_2_-induced apoptosis in PC12 cells and up to 400 µg/mL in a zebrafish model of Parkinson’s disease [48,49]. Forsythoside A was also found to upregulate the expression of Nrf2 and heme oxygenase 1 in lipopolysaccharide-stimulated BV2 microglia cells and primary microglia cells, as well as, in vivo in a liver injury model [50,51]. More importantly, it has been suggested that the number of glucose units in the phenylethanoid glycosides can correlate with the degree of Nrf2/ARE pathway activation [50,52]. Therefore, the Nrf2 activity might be selectively manipulated by the structural peculiarities of the molecules, their ratio, and is eventually associated with their physicochemical properties, such as cell permeability.

Our findings suggest that myconoside and calceolarioside E might regulate Nrf2 expression in neutrophils. The mechanism that controls the enhanced expression of Nrf2 in neutrophils, caused by myconoside and calceolarioside E is a next question to be clarified. However, the Nrf2-modulating activity of these compounds can be therapeutically exploited to balance the antioxidant homeostasis, apoptosis, cell trafficking, and homing in pathological conditions.

## 4. Conclusions

Several phenylethanoid glycosides-rich fractions have been obtained from the crude methanolic extract of *H. rhodopensis*. Their contents were characterized by NMR analysis and the two main molecules myconoside and calceolarioside E were found in different ratios in the fractions. The effect on Nrf2 expression in primary BM neutrophils of the fractions, containing specific ratios of myconoside and calceolarioside E, as well as, a single or combinatorial treatment of both molecules have been evaluated. The most active fractions were D and E, where myconoside and calceolarioside E were in ratios 1:0.6 and 0.25:1, respectively. Calceolarioside E (32 µg/mL) had a stronger activity than myconoside, the effect of which was very similar to that of CDDO-Me.

The obtained data indicate that both molecules, used alone or in combination, have a stimulatory activity on Nrf2 intracellular expression. The Nrf2 activation can be manipulated by changes in the structural peculiarities of the molecules, their ratio, and physicochemical properties, such as intracellular permeability. We strongly believe that further studies should be designed and performed to clarify the mechanism for enhanced endogenous Nrf2 level caused by myconoside and calceolarioside E, as well as, to show the compound’s selectivity towards the Nrf2 activity. Such investigations will support the therapeutic use of either the isolated compounds or the *H. rhodopensis* fractions in pathologies related to a failure/dysfunction in the Nrf2 activity or expression.

## 5. Materials and Methods

### 5.1. Materials and Reagents

Deuterated methanol (purity 99.8%) and deuterium oxide (99.9%) were supplied from Deutero GmbH (Kastellaun, Germany). Trimethyl silylpropionic acid sodium salt-*d*_4_ (TSPA-*d*_4_), acetonitrile, acetic, formic acids, methanol, and DMSO of the HPLC grade and CDDO-Me were purchased from Merck KGaA (Darmstadt, Germany). Deionized water was purified through Ultrapure Water Systems Arium^®^ 611DI (Sartorius AG, Gottingen, Germany). Myconoside and calceolarioside E were isolated and purified within the current study. The CDDO-Me and myconoside were dissolved in DMSO, while calceolarioside E was dissolved in methanol. The compounds were then diluted with PBS (pH 7.4) to a concentration of 1 mg/mL, passed through a sterile 20 µm syringe filter (Corning GmbH, Kaiserslautern, Germany), and stored at −20 °C.

Column chromatography (CC) on Polyamide 6 (Fluka, Germany), Sephadex LH-20 and Lobar chromatography (Lobar RP-18, Merck) were used for separation and purification of the individual compounds. Preparative thin layer chromatography (PTLC) was performed on pre-coated plates 60 F254, 0.25 mm and thin layer chromatography (TLC) was performed on 60 F254 plates (Merck). Separation was visualized by spraying with 20% (*v*/*v*) H_2_SO_4_ in an ethanol solution.

### 5.2. Plant Material Collection

Wild grown *H. rhodopensis* plants were kindly provided by the Center of Plant Systems Biology and Biotechnology (CPSBB). The plants were collected near Bachkovo, Bulgaria (latitude: 41°59′18.0″ N, longitude: 24°52′37.4″ E, 310 m a.s.l.) on 23 May 2020 with permission from the Ministry of Environment and Water (no. 800/08.07.2019) issued to CPSBB. The plant material was collected by Dr. Nikola Staykov from CPSBB.

### 5.3. In Vitro Cultivation of H. rhodopensis

In vitro plants of *H. rhodopensis* were grown on a plant growth regulator-free full-strength McCown Woody Plant Medium (WPM) supplemented with 2.0% sucrose and 0.6% plant agar (*w*/*v*) at 20 °C, 80% humidity, under 16 h light (30–40 μM/m^2^/s)/8 h dark regime, and were subcultured every 2 months. The medium pH was adjusted to 5.8 before autoclaving.

### 5.4. Extraction and Isolation of Pure Compounds

Air-dried and grounded plant material (13.8 g) was extracted with 500 mL MeOH (2 × 48 h). Crude MeOH extracts were combined, filtrated and evaporated (4.5 g), dissolved in 10 mL distilled H_2_O, and subjected to polyamide column chromatography eluting with 300 mL H_2_O and H_2_O–MeOH mixtures (25–100%) to give ten main fractions, A-J. Part of fraction B (0.2 g) was dissolved in water and purified using LPLC (Lobar RP-18, size A, eluents 50 mL H_2_O and 2.5–30% MeOH) followed by PTLC with CH_3_Cl:MeOH:H_2_O (61:32:7, *v*/*v*/*v*) to obtain pure myconoside (30.0 mg). Part of fraction E (38.1 mg) was subjected on PTLC similarly to afford pure calceolarioside E (6.6 mg). Fraction H (0.14 g) was separated on a Sephadex-LH20 column eluting with MeOH and a subtraction (75.5 mg) was further purified by LPLC (Lobar RP-18, size A, eluents 50 mL H2O and 25–60% MeOH) to obtain hispidulin 8-*C*-(2-*O*-syringoyl-*β*-glucopyranoside (19.5 mg) and hispidulin 8-*C*-(6-*O*-acetyl-2-*O*-syringoyl-*β*-glucopyranoside) (16.4 mg).

### 5.5. Plant Material Extraction for NMR and HPLC Analysis

The wild grown *H. rhodopensis*, as well as, 2-months-old in vitro plants were freeze-dried and extracted with 50% aqueous methanol (1:20) under sonication for 20 min at room temperature. The extracts were filtrated, concentrated under a vacuum at 40 °C, lyophilized until dryness, and stored at −20 °C before use for NMR and HPLC analysis.

### 5.6. Nuclear Magnetic Resonance (NMR) Analysis

The NMR analysis was performed according to the previously established protocol from [6,47] according to which 10 mg of the plant extracts were thoroughly homogenized in a 2 mL Eppendorf with equal amounts of 0.4 mL CD_3_OD and D_2_O, containing a KH_2_PO_4_ buffer with pH 6.0 and TSPA-*d*_4_ as an internal standard at a final concentration of 0.005% (*w*/*v*). After vortexing (for 1 min), the sample was placed in an ultrasonic bath (35 kHz) for 20 min at room temperature. The samples were further centrifuged for 20 min (at 12,000 rpm, 4 °C) and 0.8 mL of the supernatant was transferred in a 5 mm NMR tube. The ^1^H NMR and ^2^D NMR spectra (HSQC and COSY) were recorded at 25 °C on an AVII+ 600 spectrometer (Bruker, Karlsruhe, Germany) operating at a frequency of 600.01 MHz with relaxation time of 4.07 s and CD_3_OD as an internal lock.

### 5.7. HPLC-UV Analysis

Prior to the analysis, myconoside and calceolarioside E were dissolved in methanol and standard solutions from 10–200 µg/mL (for myconoside) and 5–100 µg/mL (for calceolarioside E) were prepared and filtrated through a 0.45 µm syringe filter. The plant extracts were prepared in 5 mg/mL solutions in 50% aqueous methanol.

The analyses were performed on the HPLC system, consisting of Waters binary pump, Waters dual λ absorbance detector (Waters, Milford, MA, USA) controlled by the Breeze 3.30 software. The molecules were separated on a reverse-phase Kinetex^®^ C18, 100 Å (150 × 4.6 mm, 5 μm) core-shell column (Phenomenex, Torrance, CA, USA), operating at 26 °C.

The myconoside determination was based on an HPLC protocol previously used by [25] with the following modifications. The mobile phases used were acetonitrile (phase A) and 1% aqueous acetic acid (phase B) at a flow rate of 1.0 mL/min with the following gradient: 5:95 (A:B) from 0–3 min, followed by an increase of A from 5 to 25 (3–10 min), increase of A from 25 to 60 (10–23 min), and restoring to the initial ratio 5:95 (from 23–25 min). The detection wavelength was 278 nm. Calceolarioside E was quantified at a wavelength of 330 nm using acetonitrile (phase A) and 0.1% aqueous formic acid (phase B) at a flow rate of 1.0 mL/min and gradient as follows: 5:95 (A:B) from 0–3 min, increase of A from 5 to 50 (from 3–6 min), followed by another increase from 50 to 65 (from 6–7 min) and then decrease of A from 65 to 50 (from 7 to 12 min), 50 to 20 (from 12–20 min) and turning back to the initial 5:95 (from 20–25 min).

### 5.8. Animals

BALB/c mice were purchased from Charles River Laboratories (Wilmington, MA, USA). The experiments were adapted from protocols previously described and approved by the National Food Agency (Bulgaria) according to the National and European Guidelines (License for Animal Housing no. 352/30.01.2012 (registration no. 11130005); License for Experimental Procedures no. 105/10.07.2014). All the experiments were conducted in accordance with the ARRIVE criteria (Animal Research: Reporting of In Vivo Experiments). Mice were kept under standard conditions in the Experimental Animals Facility at the Institute of Microbiology (Bulgarian Academy of Sciences, Sofia, Bulgaria), and the experiments were provided under anesthesia and the control of a veterinarian.

### 5.9. Cell Isolation and Purification

Femur and tibia were collected from female or male BALB/c mice (at the 14th week age, 17–19 g) and BM suspension was prepared following the previously described protocol [53]. Neutrophils were purified from BM suspension by immunomagnetic sorting using a MojoSort™ Mouse Neutrophil Isolation Kit and according to the manufacturer’s protocol (Biolegend, London, UK). Briefly, BM cells were counted and re-suspended at a concentration of 1 × 10^7^/mL in 100 µL of a selection buffer (5% Bovine serum albumin (BSA)/PBS containing 2 mM ethylenediaminetetraacetic acid (EDTA; Sigma-Aldrich, Darmstadt, Germany)). The antibody cocktail containing biotin-labelled antibodies against CD4, CD5, B220, CD11c, CX3CR1, F4/80, CD117, CD244.2, TER-119/Erythroid was added to the cell suspension at a volume of 10 µL. After incubation for 20 min at 4 °C, the cells were washed with 10 mL cold PBS (centrifugation at 250× *g* for 10 min) and resuspended in 100 µL of a selection buffer. Streptavidin Nanobeads at a volume of 10 µL were added to the cell suspension and incubated for 20 min at 4 °C. The cells were washed with 10 mL PBS and resuspended in a 2 mL selection buffer. The negative magnet selection was performed twice for 5 min using an Affyimetrix magnet (Thermo Fisher Scientific, Waltham, CA, USA). Purified murine neutrophils contained 77–80% Ly6G^+^CD11b^+^ cells (evaluated by flow cytometry) and were with 95% vitality (evaluated by Trypan blue dye staining).

### 5.10. Cell Culture and Treatment

Purified neutrophils were counted on a Burker chamber and resuspended at a concentration of 2 × 10^6^/mL in a sterile complete RPMI-1640 medium containing 10% Fetal calf serum (FCS), 2 mM L-glutamine, 100 U/mL penicillin, 100 µg/mL streptomycin, and supplemented with amino acids (all from Sigma-Aldrich, St. Louis, MO, USA). The cells at a concentration of 1 × 10^6^/mL were seeded on 24-well plates (Corning) in the presence of 200 μg/mL of *H. rhodopensis* extract or 200 μg/mL of fractions (named B, D, and E) or purified compounds myconoside and calceolarioside E (at concentrations corresponding to their content in the D and E fractions). Pure myconoside was used at a concentration of 32 μg/mL which was six times lower than the content of myconoside in fraction B (200 μg/mL). Control cultures were treated with DMSO (0.02%), methanol (0.01%), or a well-known Nrf2 activator—CDDO-Me (5 μM). Neutrophils were left unstimulated to determine the endogenous Nrf2 level or were stimulated with 100 ng/mL PMA. After 16 h of incubation at 37 °C, CO_2_ cells were collected, washed with 10 mL PBS, and used in the flow cytometry analysis.

### 5.11. Measuring Cell Death by the Trypan Blue Uptake

The neutrophil cell death was measured by the uptake of Trypan Blue dye (0.4% solution in PBS (pH 7.4), Sigma-Aldrich, Munich, Germany) according to the protocol described previously [50] and using light microscopy (Nikon, Amsterdam, The Netherlands). The survived cells were calculated as follows:

% viable cells = [1.00 − (Number of Trypan blue positive cells ÷ Number of total cells)] × 100(1)

### 5.12. Flow Cytometry for Evaluation of Intracellular Nrf2 Expression

Neutrophils (1 × 10^6^/mL) were resuspended in a 5% BSA/PBS buffer and were incubated for 20 min at 4 °C, in the dark, with validated concentrations of fluorochrome-conjugated antibodies against mouse Ly6G (clone 1A8, Biolegend) and CD11b (clone M1-70, Biolegend), and of the corresponding isotype rat control antibodies (Biolegend). The Antibodies were conjugated with the following fluorophores: Peridinin chlorophyll protein (PerCP) Cy5.5 and allophycocyanin (APC), respectively. The samples were washed twice with PBS and fixed with 4% paraformaldehyde (PFA)/PBS for 10 min at room temperature. After 2-folds of washing with PBS, cells were resuspended in 100 µL of a permeabilizing/blocking buffer (Biolegend) and incubated for 15 min at room temperature. Then, the cells were stained with a purified rabbit antibody against Nrf2 (1:500 diluted, Cell Signaling Technology, Leiden, The Netherlands) for 1 h at 4 °C, in the dark. After washing, the cells were stained with fluorescein isothiocyanate (FITC)-labeled anti-rabbit IgG (diluted 1:1600, Sigma-Aldrich, Munich, Germany) for 1 h at 4 °C, in the dark, washed twice, and subjected to the flow cytometry analysis.

### 5.13. Data Analysis

The obtained 1D and 2D NMR spectra were automatically reduced to ASCII files using the AMIX software (version 3.7, Bruker), phase, base line corrected, and referenced at 0.0 ppm to the internal standard TSPA using the MestReNova software (version 12.0.0, Mestrelab Research, Santiago de Compostela, Spain). All the signals were normalized in relation to the peak of TSPA and scaled to 1.0.

The flowcytometry data were analyzed after the acquisition of at least 20,000 cell counts/sample and singlet/doublet cell discrimination, run on a BSRII flow cytometer using the BD FACSDiva v6.1.2 Software (Becton Dickinson GmbH, San Jose, CA, USA) at the Department of Immunology’s Flow Cytometry Core, The Stephan Angeloff Institute of Microbiology, Sofia, Bulgaria. The viability and flowcytometry data are expressed as the mean ± standard deviation (SD). The significant differences between the groups were evaluated by the Student’s unpaired *t*-test. Statistical significance is displayed as: N.S., not significant; *p*  <  0.05.

## Data Availability

All relevant data are within the manuscript. The data set generated and analyzed during the current study also available from the corresponding author upon request.

## Abbreviations

ARE	Antioxidant responsible elements
BM	Bone marrow
CDDO-Me	Cyano-3,12-dioxo-oleana-1,9(11)-dien-28-oic acid methyl ester
DMSO	Dimethyl sulfoxide
DW	Dry weight
HSQC	Heteronuclear single quantum coherence spectroscopy
HPLC	High performance liquid chromatography
KEAP1	Kelch-like ECH-associated protein 1
NMR	Nuclear magnetic resonance
Nrf2	Nuclear factor erythroid 2 p45-related factor 2
PBS	Phosphate buffered saline
PMA	Phorbol 12-myristate 13-acetate
ROS	Reactive oxygen species
